# Supplementary figures and images for: Make a choice: A rapid strategy for minimizing peat in horticultural press pots substrates using a constrained mixture design and surface response approach
Source: PLoS One. 2023 Jul 31;18(7):e0289320. doi: 10.1371/journal.pone.0289320 (PMC10389738; doi:10.1371/journal.pone.0289320)

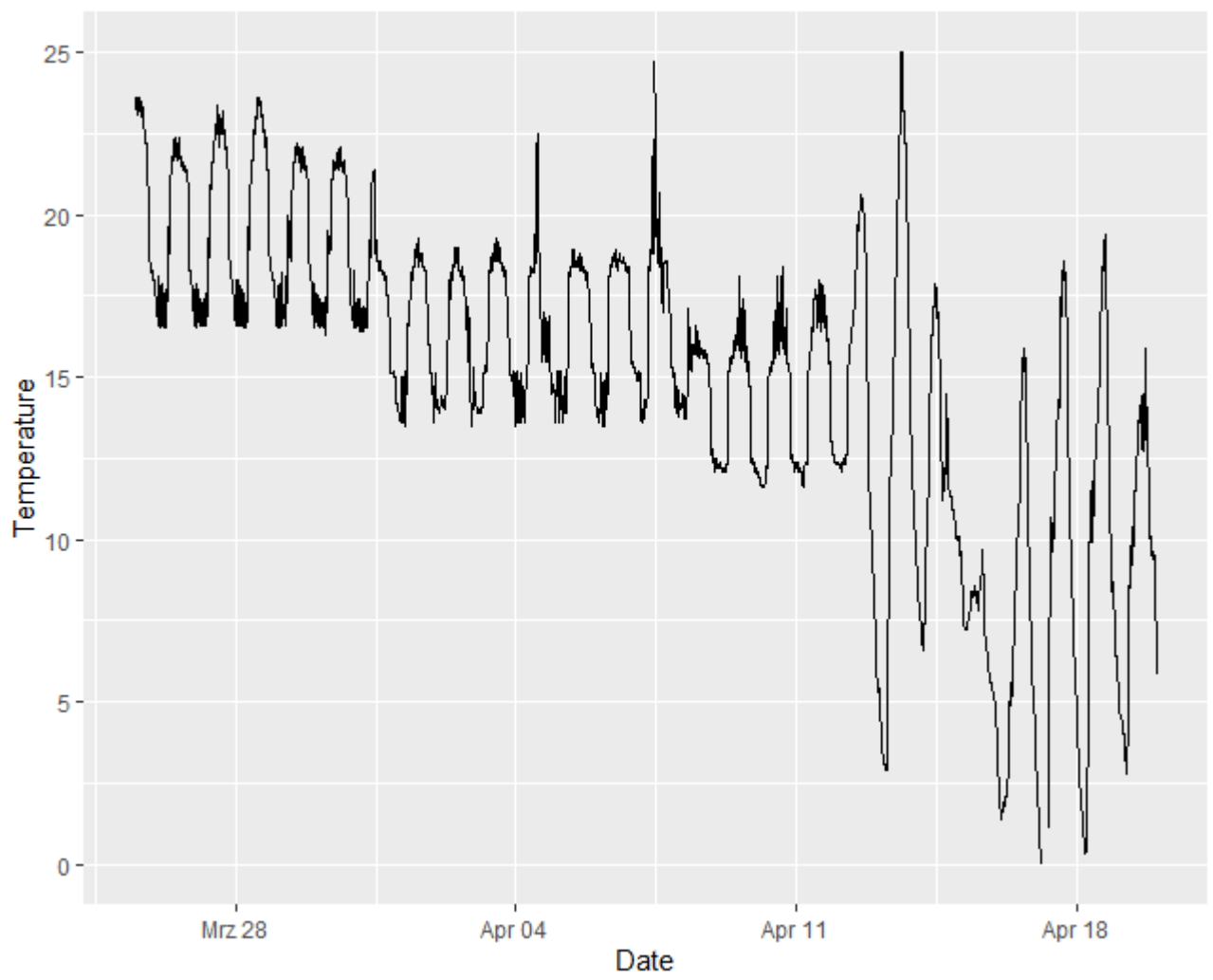

Supplement: S1 Fig — (PDF) [file pone.0289320.s001.pdf]

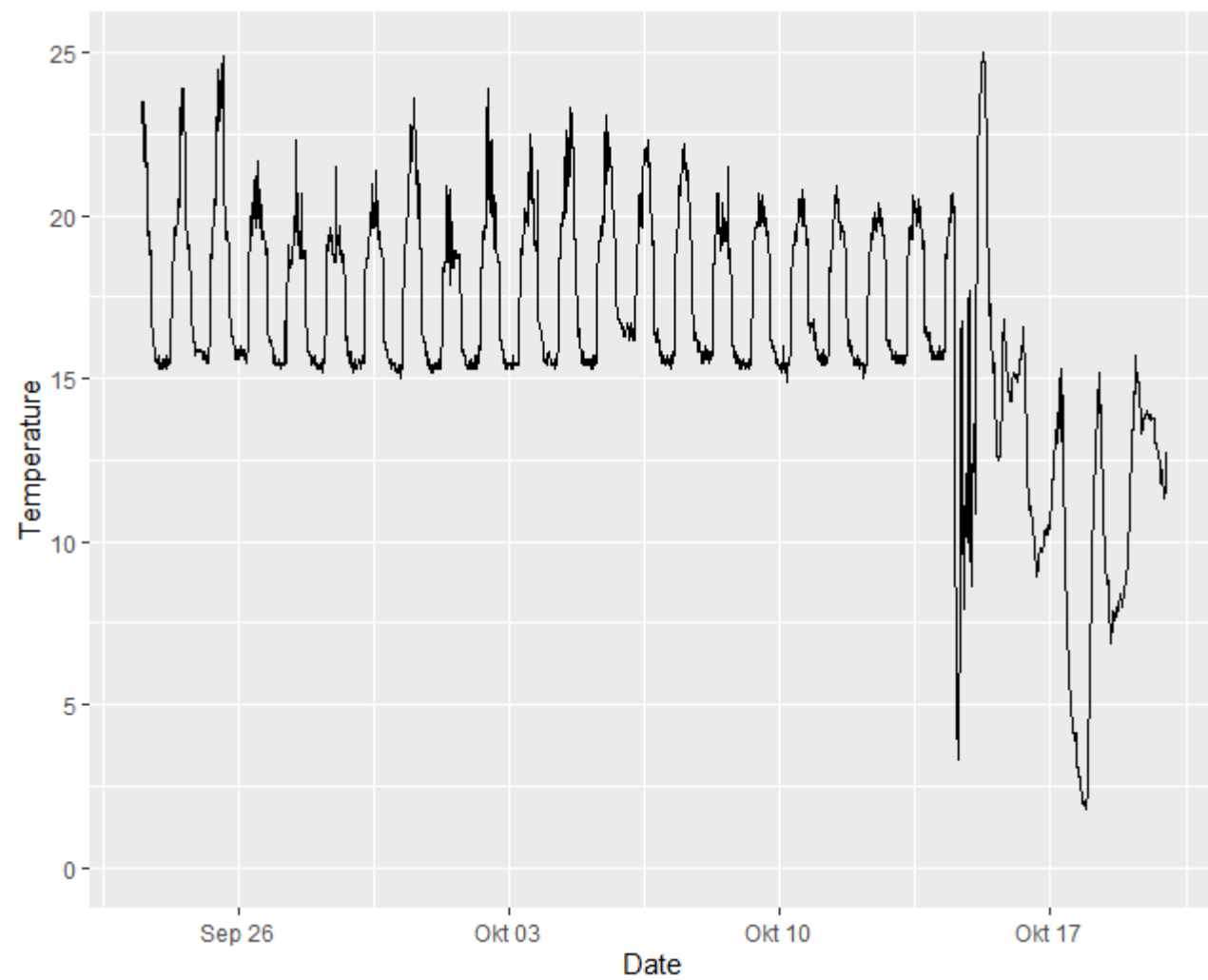

Supplement: S2 Fig — (PDF) [file pone.0289320.s002.pdf]

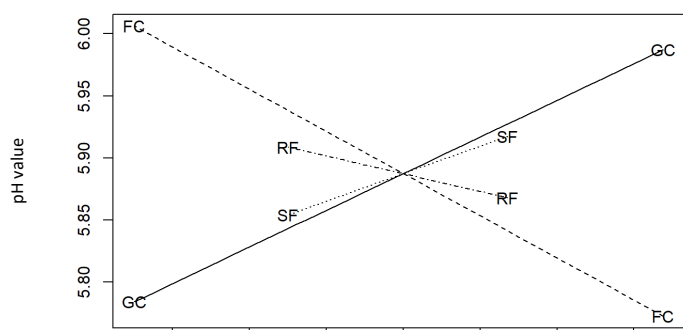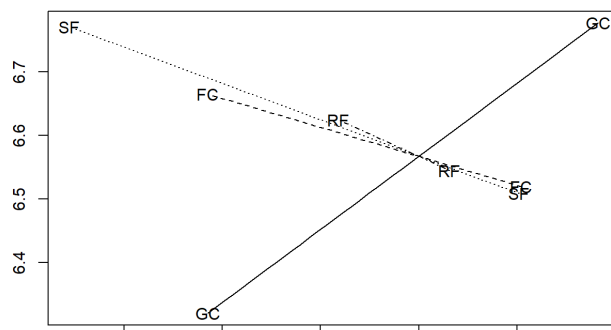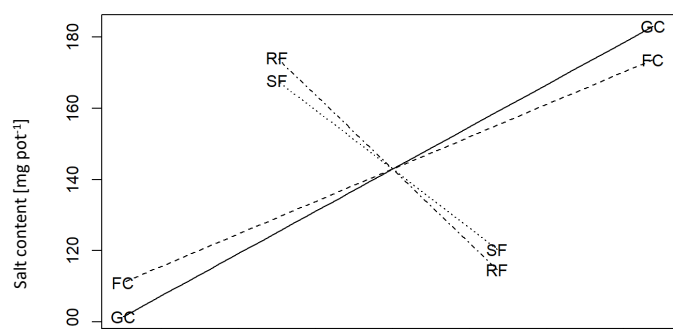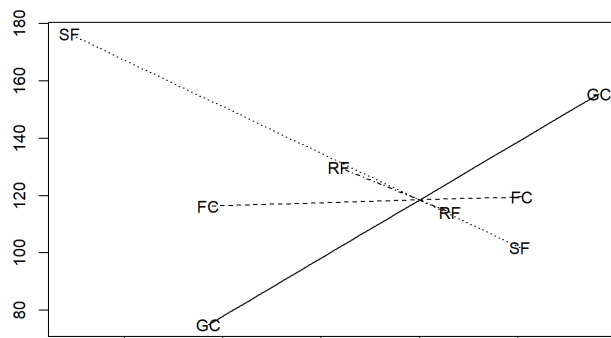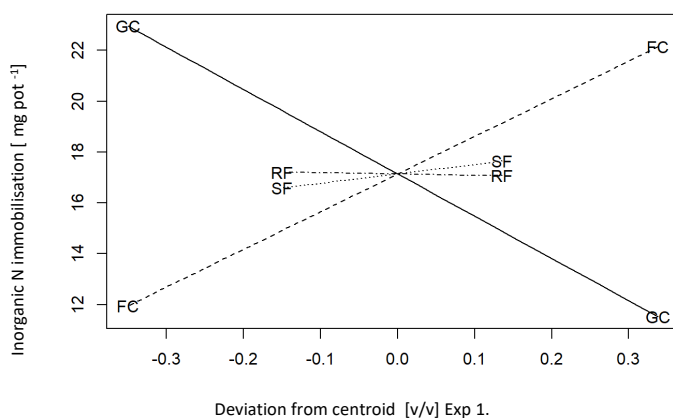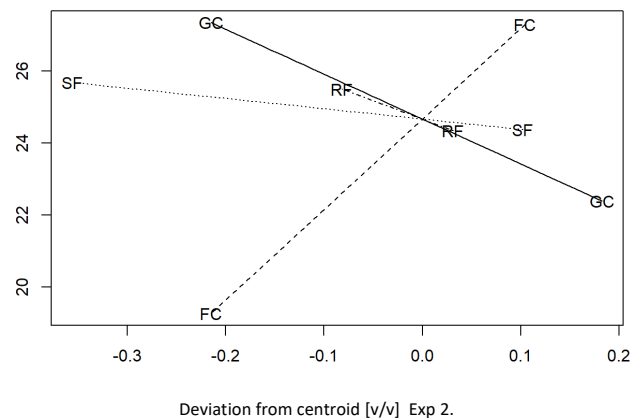

Supplement: S3 Fig — (PDF) [file pone.0289320.s003.pdf]

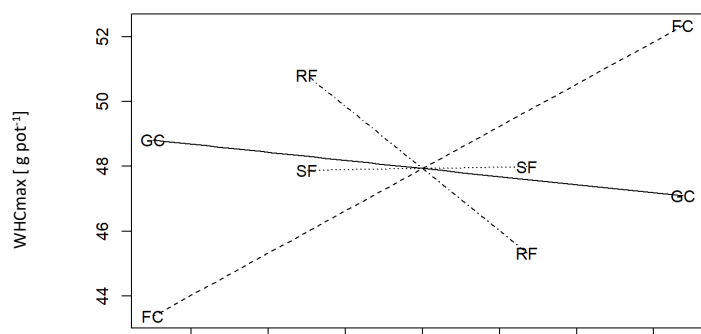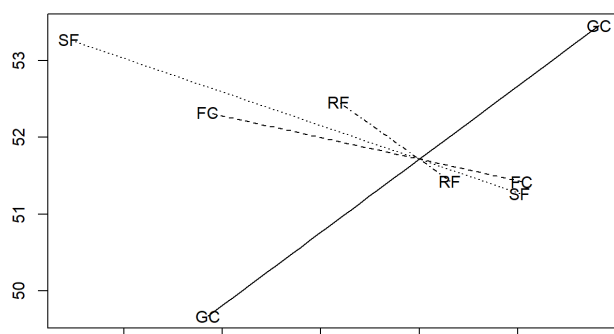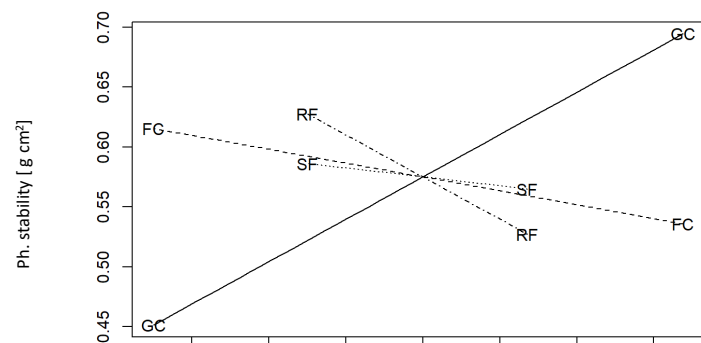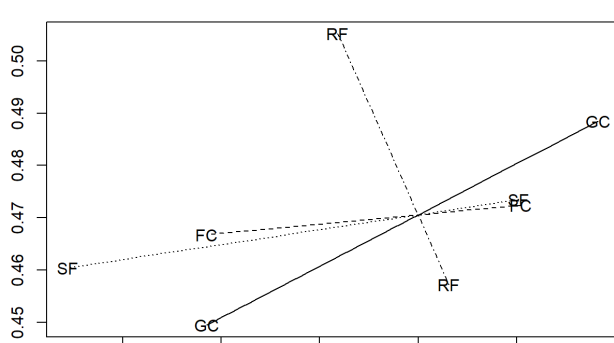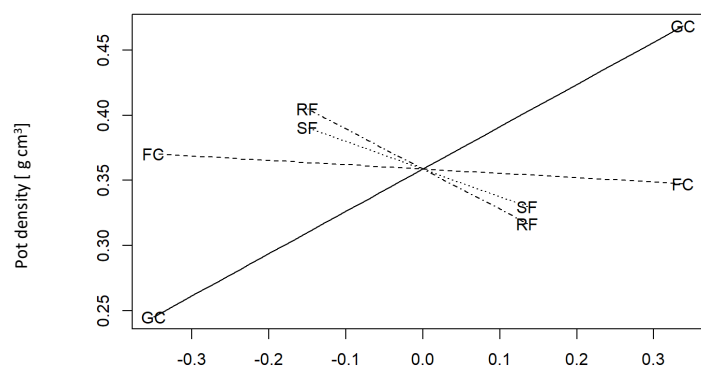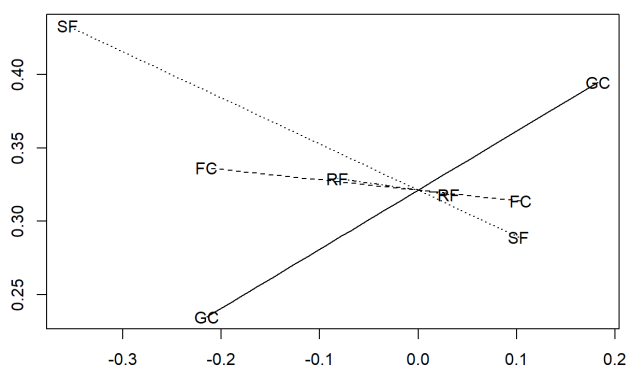

Deviation from centroid [v/v] Exp 1.

Deviation from centroid [v/v] Exp 2.

Supplement: S4 Fig — (PDF) [file pone.0289320.s004.pdf]
