# Supplementary material for: Make a choice: A rapid strategy for minimizing peat in horticultural press pots substrates using a constrained mixture design and surface response approach
Source: PLoS One. 2023 Jul 31;18(7):e0289320. doi: 10.1371/journal.pone.0289320 (PMC10389738; doi:10.1371/journal.pone.0289320)
Supplement: S1 Table — (PDF) [file pone.0289320.s005.pdf]

MIX	GC	FC	SF	RF	Peat	Fresh plant biomass		WHCmax		Pot density		pH		Stability	Volume
	[% v/v]					g pot $^{-1}$				g DM cm^{-3}				kg cm^{-2}	cm^{3}
						**17 DaS**	**25 DaS**	**4 DaS**	**25 DaS**	**4 DaS**	**25 DaS**	**4 DaS**	**25 DaS**	**25 DaS**	**25 DaS**
						Exp. 1 (50% v/v)									
**M 1**	**35**	**15**	**0**	**0**	**50**	0.77 (0.10)	2.04 (0.25)	49.32 (2.33)	39.24 (4.50)	0.48 (0.03)	0.47 (0.03)	5.96 (0.07)	6.25 (0.09)	0.72 (0.03)	52.42 (2.03)
**M 2**	**35**	**0**	**15**	**0**	**50**	0.73 (0.10)	2.22 (0.22)	45.82 (0.66)	36.09 (2.91)	0.46 (0.03)	0.42 (0.04)	6.03 (0.02)	6.37 (0.02)	0.70 (0.06)	54.08 (6.34)
**M 3**	**35**	**0**	**0**	**15**	**50**	0.82 (0.14)	2.39 (0.14)	42.18 (0.67)	39.17 (2.80)	0.44 (0.04)	0.43 (0.03)	6.01 (0.01)	6.31 (0.08)	0.77 (0.10)	54.56 (2.88)
**M 4**	**0**	**35**	**15**	**0**	**50**	0.74 (0.11)	2.61 (0.34)	54.42 (2.23)	50.48 (2.09)	0.29 (0.02)	0.27 (0.01)	5.82 (0.02)	6.18 (0.06)	0.57 (0.06)	63.14 (2.53)
**M 5**	**0**	**35**	**0**	**15**	**50**	0.85 (0.12)	2.57 (0.31)	48.07 (1.35)	46.71 (2.74)	0.26 (0.02)	0.27 (0.01)	5.66 (0.05)	6.07 (0.19)	0.39 (0.12)	60.58 (3.67)
**M 6**	**15**	**35**	**0**	**0**	**50**	0.70 (0.13)	2.47 (0.35)	52.78 (2.21)	45.23 (2.52)	0.43 (0.05)	0.38 (0.03)	5.77 (0.04)	6.06 (0.02)	0.64 (0.06)	58.66 (3.30)
**M 7**	**20**	**20**	**5**	**5**	**50**	0.88 (0.17)	2.67 (0.41)	50.95 (1.10)	45.94 (2.19)	0.39 (0.02)	0.38 (0.01)	5.92 (0.02)	6.06 (0.05)	0.53 (0.06)	60.99 (2.47)
**C**	**0**	**0**	**0**	**0**	**100**	0.85 (0.07)	2.82 (0.39)	52.93 (1.58)	48.03 (3.79)	0.22 (0.01)	0.23 (0.01)	5.88 (0.02)	5.69 (0.09)	0.60 (0.04)	53.48 (4.39)
CD						0.26	0.61	3.32	5.85	0.067	0.049	0.075	0.18	0.145	7.17
						**18 DaS**	**26 DaS**	**4 DaS**	**26 DaS**	**4 DaS**	**26 DaS**	**4 DaS**	**26 DaS**	**26 DaS**	**26 DaS**
						Exp. 2 (25% v/v)									
**M 1**	**40**	**0**	**35**	**0**	**25**	0.96 (0.34)	3.31 (0.69)	53.67 (6.00)	51.44 (2.69)	0.36 (0.05)	0.39 (0.02)	6.86 (0.03)	6.75 (0.04)	0.52 (0.07)	74.98 (2.42)
**M 2**	**40**	**25**	**10**	**0**	**25**	0.78 (0.26)	3.70 (0.47)	54.15 (4.25)	45.81 (2.27)	0.44 (0.03)	0.36 (0.02)	6.76 (0.02)	6.64 (0.07)	0.54 (0.07)	76.77 (2.19)
**M 3**	**40**	**25**	**0**	**10**	**25**	0.65 (0.10)	3.41 (0.49)	52.08 (2.56)	53.10 (3.10)	0.41 (0.02)	0.41 (0.02)	6.81 (0.03)	6.58 (0.09)	0.44 (0.06)	80.10 (4.92)
**M 4**	**10**	**25**	**35**	**5**	**25**	1.16 (0.28)	3.85 (0.55)	54.18 (2.74)	47.06 (5.51)	0.25 (0.03)	0.24 (0.02)	6.37 (0.02)	6.70 (0.02)	0.50 (0.06)	78.30 (2.91)
**M 5**	**15**	**25**	**35**	**0**	**25**	1.15 (0.30)	3.97 (0.58)	44.17 (2.08)	48.94 (3.78)	0.24 (0.02)	0.24 (0.02)	6.44 (0.03)	6.73 (0.03)	0.45 (0.06)	81.92 (3.03)
**M 6**	**40**	**0**	**25**	**10**	**25**	0.96 (0.20)	3.86 (0.56)	54.55 (5.45)	48.77 (3.78)	0.39 (0.05)	0.33 (0.01)	6.80 (0.02)	6.80 (0.06)	0.45 (0.06)	81.15 (4.05)
**M 7**	**10**	**25**	**30**	**10**	**25**	1.01 (0.25)	3.82 (0.35)	53.94 (3.26)	48.85 (3.56)	0.25 (0.02)	0.24 (0.02)	6.32 (0.03)	6.71 (0.02)	0.46 (0.06)	79.68 (3.03)
**M 8**	**35**	**0**	**35**	**5**	**25**	0.81 (0.22)	3.11 (0.57)	54.13 (3.82)	47.31 (3.77)	0.34 (0.02)	0.31 (0.02)	6.67 (0.04)	6.70 (0.03)	0.51 (0.06)	77.98 (1.78)
**M 9**	**35**	**0**	**30**	**10**	**25**	0.94 (0.16)	3.02 (0.49)	53.89 (1.81)	45.11 (5.86)	0.35 (0.04)	0.30 (0.03)	6.65 (0.05)	6.73 (0.02)	0.45 (0.05)	80.93 (6.83)
**M 10**	**29**	**14**	**26**	**6**	**25**	1.06 (0.16)	4.20 (0.52)	52.31 (1.91)	53.98 (5.56)	0.34 (0.04)	0.33 (0.02)	6.50 (0.08)	6.75 (0.10)	0.43 (0.07)	83.10 (4.90)
**C**	**0**	**0**	**0**	**0**	**100**	1.04 (0.21)	3.37 (0.46)	68.66 (1.60)	58.66 (6.06)	0.22 (0.02)	0.21 (0.02)	5.94 (0.02)	5.68 (0.06)	0.41 (0.02)	77.66 (2.06)
CD						0.50	1.13	11.95	14.26	0.072	0.046	0.085	0.115	0.125	8.25
