# Supplementary material for: Make a choice: A rapid strategy for minimizing peat in horticultural press pots substrates using a constrained mixture design and surface response approach
Source: PLoS One. 2023 Jul 31;18(7):e0289320. doi: 10.1371/journal.pone.0289320 (PMC10389738; doi:10.1371/journal.pone.0289320)
Supplement: S2 Table — (PDF) [file pone.0289320.s006.pdf]

MIX	GC	FC	SF	RF	Peat	N_{min}-N			NH_4-N			N_{min}-N reduction		Salt content	
	[% v/v]					mg pot $^{-1}$									
						**4 DaS**	**17 DaS**	**25 DaS**	**4 DaS**	**17 DaS**	**25 DaS**	**17 DaS**	**25 DaS**	**4 DaS**	**25 DaS**
						Exp. 1 (50% peat)									
**M 1**	**35**	**15**	**0**	**0**	**50**	18.03 (4.48)	14.38 (3.65)	7.35 (3.76)	2.79 (0.56)	0.07 (0.04)	0.10 (0.03)	3.65 (3.65)	10.67 (3.76)	205.55 (23.68)	119.87 (15.18)
**M 2**	**35**	**0**	**15**	**0**	**50**	10.95 (1.06)	4.83 (2.22)	0.19 (0.12)	1.00 (0.29)	0.03 (0.04)	0.13 (0.08)	6.12 (2.22)	10.77 (0.12)	147.10 (7.72)	99.54 (25.75)
**M 3**	**35**	**0**	**0**	**15**	**50**	11.10 (1.85)	10.23 (2.39)	1.25 (0.53)	2.15 (0.46)	0.02 (0.03)	0.07 (0.04)	0.87 (2.39)	9.85 (0.53)	146.76 (12.46)	106.71 (25.28)
**M 4**	**0**	**35**	**15**	**0**	**50**	26.78 (5.26)	8.41 (4.47)	2.49 (1.75)	1.51 (0.65)	0.03 (0.03)	0.17 (0.03)	18.36 (4.47)	24.28 (1.75)	142.00 (19.79)	82.92 (28.68)
**M 5**	**0**	**35**	**0**	**15**	**50**	25.48 (1.81)	13.45 (3.41)	2.41 (0.79)	3.68 (0.41)	0.08 (0.04)	0.11 (0.03)	12.04 (3.41)	23.07 (0.79)	120.80 (25.76)	100.00 (33.82)
**M 6**	**15**	**35**	**0**	**0**	**50**	30.74 (4.86)	17.00 (4.77)	8.60 (2.82)	4.84 (1.51)	0.08 (0.07)	0.12 (0.05)	13.74 (4.77)	22.13 (2.82)	208.81 (12.36)	149.29 (11.37)
**M 7**	**20**	**20**	**5**	**5**	**50**	21.65 (0.86)	10.50 (1.72)	3.82 (1.54)	2.63 (0.12)	0.07 (0.03)	0.12 (0.04)	11.15 (1.72)	17.83 (1.54)	188.17 (11.89)	103.68 (12.31)
**C**	**0**	**0**	**0**	**0**	**100**	19.14 (1.20)	10.02 (3.68)	2.92 (0.78)	11.77 (0.69)	7.11 (2.29)	2.85 (0.73)	9.13 (3.68)	16.22 (0.78)	127.41 (5.46)	107.39 (19.48)
CD						6.78	6.83	4.04	1.42	0.085	0.095	6.83	4.04	34.95	46.62
						**4 DaS**	**18 DaS**	**26 DaS**	**4 DaS**	**18 DaS**	**26 DaS**	**18 DaS**	**26 DaS**	**4 DaS**	**26 DaS**
						Exp. 2 (25% peat)									
**M 1**	**40**	**0**	**35**	**0**	**25**	10.65 (2.54)	6.68 (1.64)	0.25 (0.08)	9.39 (2.21)	0.16 (0.03)	0.17 (0.05)	8.98 (1.64)	15.40 (0.08)	142.70 (21.79)	115.85 (22.84)
**M 2**	**40**	**25**	**10**	**0**	**25**	27.53 (4.85)	24.63 (5.56)	8.61 (6.23)	11.99 (1.45)	0.11 (0.04)	0.10 (0.05)	7.90 (5.56)	23.92 (6.23)	189.80 (6.57)	122.47 (34.17)
**M 3**	**40**	**25**	**0**	**10**	**25**	27.59 (5.96)	29.20 (3.63)	13.72 (3.04)	12.39 (2.48)	0.55 (0.40)	0.11 (0.03)	3.39 (3.63)	18.87 (3.04)	168.04 (20.52)	140.98 (40.13)
**M 4**	**10**	**25**	**35**	**5**	**25**	19.04 (2.74)	7.60 (1.15)	0.24 (0.06)	4.24 (0.33)	0.52 (0.28)	0.19 (0.04)	16.44 (1.15)	23.79 (0.06)	88.26 (9.80)	43.50 (21.59)
**M 5**	**15**	**25**	**35**	**0**	**25**	17.06 (4.27)	7.33 (1.47)	0.45 (0.36)	4.61 (1.07)	0.46 (0.23)	0.20 (0.04)	14.73 (1.47)	21.61 (0.36)	82.86 (7.95)	40.75 (12.90)
**M 6**	**40**	**0**	**25**	**10**	**25**	10.02 (1.38)	7.61 (1.65)	0.36 (0.17)	8.26 (1.28)	0.26 (0.17)	0.14 (0.06)	7.41 (1.65)	14.66 (0.17)	128.61 (10.39)	95.62 (44.00)
**M 7**	**10**	**25**	**30**	**10**	**25**	21.10 (3.84)	6.92 (2.00)	0.38 (0.14)	4.45 (0.37)	0.49 (0.22)	0.22 (0.06)	19.18 (2.00)	25.73 (0.14)	87.31 (12.80)	43.64 (16.68)
**M 8**	**35**	**0**	**35**	**5**	**25**	8.87 (1.79)	4.64 (0.62)	0.28 (0.09)	6.95 (1.56)	0.25 (0.07)	0.13 (0.03)	9.23 (0.62)	13.59 (0.09)	123.95 (3.71)	98.58 (11.46)
**M 9**	**35**	**0**	**30**	**10**	**25**	8.51 (2.11)	5.01 (2.17)	0.23 (0.11)	5.85 (1.11)	0.53 (0.47)	0.11 (0.05)	8.51 (2.17)	13.28 (0.11)	129.18 (6.73)	64.53 (17.84)
**M 10**	**29**	**14**	**26**	**6**	**25**	12.52 (2.61)	8.12 (2.52)	0.30 (0.19)	5.55 (0.40)	0.36 (0.21)	0.14 (0.06)	9.40 (2.52)	17.23 (0.19)	116.68 (19.84)	72.83 (18.29)
**C**	**0**	**0**	**0**	**0**	**100**	20.07 (5.99)	12.78 (2.21)	4.85 (2.80)	17.12 (3.58)	12.30 (1.90)	4.79 (2.80)	7.28 (2.21)	15.21 (2.80)	114.08 (7.17)	83.27 (27.15)
CD						7.40	5.54	4.66	2.99	0.535	0.100	5.54	4.65	28.58	44.70
