# Supplementary material for: Make a choice: A rapid strategy for minimizing peat in horticultural press pots substrates using a constrained mixture design and surface response approach
Source: PLoS One. 2023 Jul 31;18(7):e0289320. doi: 10.1371/journal.pone.0289320 (PMC10389738; doi:10.1371/journal.pone.0289320)
Supplement: S3 Table — Root mean squared error (RMSE), corrected multiple R-squared (R2), ratio of performance to deviation (RPD). Significance level of estimate: ** = P < 0.001; * = P < 0.05. (PDF) [file pone.0289320.s007.pdf]

	Plant biomass		WHCmax		Salt content		Pot density	
	g pot $^{-1}$				mg pot $^{-1}$		g DM cm^{-3}	
	**17** DaS	**25** DaS	**4** DaS	**25** DaS	**4** DaS	**25** DaS	**4** DaS	**25** DaS
				Exp. 1: 50 % peat				
GC	0.75	2.0	46.3**	34.9**	220**	136**	0.57**	0.54**
FC	0.75	2.5	56.4**	50.5**	202**	132**	0.34**	0.31**
SF	0.75	2.8	48.3**	46.2**	2	-11	0.18**	0.17**
RF	1.09	3.0	31.6**	45.0**	-34	30	0.10*	0.18**
**RMSE**	0.12	0.30	1.75	3.11	18.77	22.80	0.03	0.02
**R^2**	0.12	0.24	0.82	0.67	0.72	0.36	0.86	0.91
**RPD**	1.08	1.16	2.40	1.77	1.93	1.26	2.72	3.38
	**18** DaS	**26** DaS	**4** DaS	**26** DaS	**4** DaS	**26** DaS	**4** DaS	**26** DaS
				Exp. 2: 25 % peat				
GC	0.51	3.1	57.9	44.8	251**	198**	0.58**	0.53**
FC	0.97	4.6	49.5	49.9	126**	66**	0.27**	0.26**
SF	1.48	3.7	48.9	52.0	14	-18	0.12**	0.14**
RF	0.73	3.3	43.3	36.8	-9	-37	0.23**	0.15*
**RMSE**	0.23	0.56	4.10	4.61	14.85	23.51	0.03	0.03
**R^2**	0.26	0.13	0.13	0.02	0.82	0.62	0.80	0.78
**RPD**	1.17	1.08	1.09	1.02	2.40	1.63	2.25	2.15
