# Supplementary material for: Make a choice: A rapid strategy for minimizing peat in horticultural press pots substrates using a constrained mixture design and surface response approach
Source: PLoS One. 2023 Jul 31;18(7):e0289320. doi: 10.1371/journal.pone.0289320 (PMC10389738; doi:10.1371/journal.pone.0289320)
Supplement: S4 Table — Root mean squared error (RMSE), corrected multiple R-squared (R2), ratio of performance to deviation (RPD). Significance level of estimate: ** = P < 0.001; * = P < 0.05. (PDF) [file pone.0289320.s008.pdf]

	pH		Stability kg cm^{-2}	Volume cm^3	Nmin N reduction		Nmin N mg pot^{-1}		
	**4 DaS**	**25 DaS**	**25 DaS**	**25 DaS**	**17 DaS**	**25 DaS**	**4 DaS**	**17 DaS**	**25 DaS**
Exp. 1 (50 % peat)									
GC	6.08**	6.30**	0.83**	50.35	0.0	6.3**	13.0**	13.0**	6.7**
FC	5.67**	5.97**	0.49**	61.79	17.9**	26.8**	36.0**	18.1**	9.3**
SF	6.08**	6.52**	0.52**	66.31	20.8**	20.3**	6.1	-14.7**	-14.1**
RF	5.77**	6.24**	0.33*	62.85	1.5	16.7**	4.2	2.7	-12.5**
**RMSE**	0.05	0.10	0.09	3.53	3.25	2.06	3.22	3.07	1.82
**R^2**	0.87	0.54	0.58	0.49	0.75	0.89	0.83	0.60	0.72
**RPD**	2.80	1.50	1.56	1.42	1.91	3.04	2.44	1.60	1.91
	**4 DaS**	**26 DaS**	**26 DaS**	**26 DaS**	**18 DaS**	**26 DaS**	**4 DaS**	**18 DaS**	**26 DaS**
Exp. 2 (25 % peat)									
GC	7.31**	6.64	0.53**	75.89	-0.5	16.5**	19.2**	29.8**	12.7**
FC	6.21**	6.52	0.48**	80.00	22.8**	44.4**	49.4**	36.6**	14.9**
SF	6.20**	6.88	0.49**	78.90	29.1**	22.8**	-2.6	-21.7**	-15.5**
RF	5.92**	6.73	0.08	99.89	18.6**	15.3**	7.0	-1.6	1.7
**RMSE**	0.06	0.06	0.06	4.01	2.67	2.53	3.55	2.63	2.30
**R^2**	0.89	0.38	0.13	0.09	0.73	0.73	0.78	0.90	0.78
**RPD**	3.11	1.28	1.09	1.06	1.93	1.93	2.17	3.27	2.15
